# Is dry skin a clue to diabetic retinopathy? Clinical insights from the overall dry skin score

**DOI:** 10.3389/fendo.2026.1871805

**Published:** 2026-06-17

**Authors:** Özge Özer, Dilek Menteşoğlu, Göknur Yorulmaz

**Affiliations:** 1 Department of Endocrinology and Metabolism, Medical Point Hospital, İzmir University of Economics, İzmir, Türkiye; 2 Department of Dermatology and Venereology, Ankara Etlik City Hospital, Ankara, Türkiye; 3 Department of Endocrinology and Metabolism, Eskişehir Osmangazi University, School of Medicine, Eskişehir, Türkiye

**Keywords:** complications, diabetes mellitus, diabetic retinopathy, overall dry skin score, skin dryness, xerosis

## Abstract

**Background:**

Fluctuations in blood sugar and changes in water retention mechanisms in diabetes mellitus often cause dry skin in patients with diabetes. However, the impact of diabetes on skin health has often been overlooked in clinical practice.

**Aims:**

This study aimed to investigate the relationship between diabetic complications and skin dryness as measured by the Overall Dry Skin (ODS) score.

**Methods:**

This single-center cross-sectional study included 100 patients with type 2 diabetes mellitus. Skin dryness was assessed on the forearm and lower leg using the ODS score, and diabetic complications were abstracted from medical records. Group comparisons and multivariable analyses were performed to identify factors independently associated with total ODS score.

**Results:**

The forearm, lower leg, and total ODS scores were significantly higher in patients with diabetic complications compared with those without complications (p < 0.05). The forearm ODS score was significantly higher in patients with retinopathy (p = 0.003) and peripheral polyneuropathy (p = 0.017). The ODS lower leg score was significantly higher in patients with retinopathy (p = 0.007). The total ODS score was significantly higher in patients with retinopathy (p = 0.002), nephropathy (p = 0.047), and peripheral polyneuropathy (p = 0.027). In multivariable analysis, only age (p = 0.002) and retinopathy (p = 0.042) were independently associated with total ODS score.

**Conclusions:**

These findings suggest that the Overall Dry Skin score may serve as a simple, non-invasive adjunctive clinical tool for identifying patients at higher risk of diabetic retinopathy in routine clinical practice. Prospective, multicenter studies with larger sample sizes are warranted to confirm the predictive value of ODS scoring.

## 1 Introduction

Diabetes mellitus has become a growing health problem worldwide. This chronic metabolic disease affects the body’s ability to regulate blood sugar levels, leading to various complications. (1) Chronic complications of diabetes can be classified as microvascular (retinopathy, nephropathy, neuropathy) and macrovascular (cerebrovascular disease, peripheral artery disease, atherosclerotic cardiovascular disease). The common symptoms of diabetes include fatigue, excessive thirst, frequent urination, and weight changes. However, the effects of diabetes on the skin are often overlooked. (2, 3).

Fluctuations in blood sugar levels and changes in the body’s water retention mechanisms caused by diabetes can disrupt the skin’s natural moisture balance, frequently leading to dry skin in individuals with diabetes. (4) Diabetic dermopathy is a common skin condition among diabetic patients, typically occurring on the lower legs, characterized by light brown or red scaly patches that may be slightly raised or sunken. (5) Dry skin not only causes discomfort but also weakens the skin barrier, increasing the risk of infection and contributing to the development of ulcers. (6) Therefore, it is essential to prioritize skin health while managing diabetes.

Various assessment tools, such as the Overall Dry Skin (ODS) scoring system, can evaluate skin dryness objectively, enabling clinicians to determine its severity before it becomes symptomatic. (7) The ODS score is a semi-quantitative, clinician-rated scale that grades dryness from 0 (absent) to 4 (severe roughness, large scales, redness, and cracks), encompassing all major and minor signs of xerosis or ichthyosis. (8, 9) Its non-invasive nature and ease of application make it particularly suited for busy outpatient settings where laboratory-based risk stratification may be limited.

Skin dryness has been associated with microvascular complications in diabetes, yet the specific relationship between objectively measured skin dryness and diabetic retinopathy remains understudied. This study aimed to investigate the relationship between diabetic complications and skin dryness in patients with type 2 diabetes mellitus, as measured by the ODS score, with particular attention to the clinical utility of this simple assessment in identifying patients at risk for retinopathy.

## 2 Materials and methods

### 2.1 Study design and participants

This single-center, cross-sectional study enrolled 100 patients with type 2 diabetes mellitus who were seeking treatment at the Department of Endocrinology and Metabolic Diseases between September 2022 and April 2023. The study was conducted in accordance with the Declaration of Helsinki, and all patients provided written informed consent. Ethical approval was granted by the Ethics Committee of Eskişehir Osmangazi University (Approval No: E-25403353-050.99-393143).

Exclusion criteria included: age under 18 years; other potential causes of neuropathy (end-stage renal disease, alcohol abuse, vitamin B12 deficiency, or malignancy); thyroid disease; use of medications that may affect sweating (corticosteroids, antihistamines, or psychoactive drugs); and primary skin diseases (neurodermatitis, psoriasis, scleroderma, metal allergy, Raynaud syndrome, or acrocyanosis).

### 2.2 Overall dry skin score assessment

The Overall Dry Skin Score (ODS) system was used to assess skin dryness on two standardized anatomical sites: the forearm and the lower leg. The ODS is a five-point ordinal scale (0–4) that encompasses all major and minor clinical signs of dry skin (xerosis) or ichthyosis: (8, 9).

- 0 = absent.
- 1 = slight scaling, faint roughness, and dull appearance.
- 2 = small scales with a few larger scales, slight roughness, whitish appearance.
- 3 = small and larger scales evenly distributed, definite roughness, possibly slight redness, and a few superficial cracks.
- 4 = dominated by large scales, severe roughness, redness present, eczematous changes, and cracks.

ODS scoring was performed by a single trained assessor (D.M.) through direct physical examination of all enrolled patients during their outpatient visit. The assessor was a dermatologist with prior training in xerosis grading. All patients were physically examined for ODS assessment; the scoring was not based on self-report or medical records alone. While interrater reliability (e.g., intraclass correlation coefficient or Cohen’s kappa) was not formally assessed in this study — a methodological limitation acknowledged — the use of a single trained rater minimizes intra-study variability. Future multicenter studies should include formal interrater reliability evaluation. The total ODS score was calculated as the sum of forearm and lower leg scores (range 0–8).

ODS assessment was performed on the forearm and lower leg to ensure standardized and reproducible scoring, as these sites are most consistently referenced in the ODS validation literature and allow direct comparison with prior studies. Foot ODS assessment was not performed in the present study; we acknowledge that foot xerosis assessment using site-specific tools (e.g., the indicator plaster method) may be more clinically relevant for diabetic foot ulcer risk stratification and represents an important direction for future research.

### 2.3 Assessment of diabetic complications

Diabetic retinopathy was diagnosed based on ophthalmologic examination documented in patient records. Although retinopathy severity staging (nonproliferative vs. proliferative) was not consistently available for all patients — precluding dose-response analysis — retinopathy diagnosis was confirmed in all cases by a qualified ophthalmologist. Diabetic nephropathy was defined by the presence of albuminuria and/or reduced estimated glomerular filtration rate (eGFR). Peripheral neuropathy was identified based on clinical neurological evaluation documented in medical records. Macrovascular complications were determined through documented clinical history.

### 2.4 Statistical analysis

Statistical analyses were performed using NCSS 2007 (NCSS LLC, Kaysville, UT, USA). Continuous variables were assessed for normality using visual inspection of histograms and the Shapiro–Wilk test. Normally distributed variables were compared using the independent samples t-test; non-normally distributed variables were analyzed using the Mann–Whitney U test. The ODS score is an ordinal variable; therefore, nonparametric tests were preferentially applied when distributional assumptions were not met.

Multivariable linear regression analysis was performed to explore factors associated with total ODS score, given the continuous and bounded nature of the total ODS score (range 0–8) and the exploratory objective of quantifying the magnitude of association. Although ordinal logistic regression and count-based models (e.g., Poisson or negative binomial regression) represent theoretically appropriate alternatives for ordinal or count outcomes, linear regression was selected for interpretability and because residual diagnostics confirmed approximate normality and homoscedasticity. Independent variables were entered using standard binary coding (0 = absent, 1 = present for complications). Model diagnostics included assessment of collinearity and inspection of residuals. To address multicollinearity, the composite ‘presence of complications’ variable was removed from the final model; only individual complication terms and covariates were retained (see **Table 1**). Given the exploratory nature of the study and the limited sample size, no adjustment for multiple comparisons (e.g., Bonferroni correction) was applied; p-values close to the significance threshold should be interpreted with caution and considered hypothesis-generating rather than confirmatory.

**Table 1:** Multivariable linear regression: factors associated with total ODS score.

Variable	β	T	P	95% CI lower	95% CI upper
Presence of complications	−0.069	−0.451	0.653	−10.301	0.820
**Age†**	0.350	3.024	0.002*	0.020	0.082
ASCVD	0.117	0.970	0.335	−0.455	1.322
**Retinopathy†**	0.221	2.063	0.042*	0.038	2.061
Nephropathy	−0.062	−0.570	0.570	−1.400	0.776
Peripheral polyneuropathy	−0.082	−0.577	0.566	−1.295	0.713
Gender	−0.055	−0.514	0.609	−0.909	0.536
Smoking	0.021	0.207	0.836	−0.419	0.516
Alcohol use	−0.063	−0.606	0.546	−1.363	0.726
Diabetes duration	0.037	0.320	0.750	−0.043	0.059
HbA1c level	−0.071	−0.712	0.478	−0.230	0.109
Use of moisturizer	−0.105	−0.968	0.336	−1.522	0.526

The ‘Presence of complications’ composite variable was excluded from the final model due to multicollinearity with individual complication terms. The model presented includes only individual complication variables and covariates.Bold values indicate statistically significant results (p < 0.05).

A *post-hoc* power calculation (G*Power 3.1) based on the observed effect size for the retinopathy–ODS association indicated approximately 72% power at alpha = 0.05, falling below the conventional 80% threshold. Findings related to retinopathy should therefore be considered exploratory and require replication in larger, prospective cohorts.

## 3 Results

### 3.1 Baseline characteristics

patients (15%) were smokers, and 13 (13%) consumed alcohol. Fifty-eight patients (58%) had hypertension, and 40 (40%) had hyperlipidemia. Mean BMI was 30.05 ± 6.13 kg/m². Detailed baseline demographic and clinical characteristics, including skincare habits (moisturizer use: 21%) and ocular lubricant use (8%), are described above. Socioeconomic and occupational data were not systematically collected, which is acknowledged as a limitation in Section 5.

Regarding antidiabetic treatment, 38 patients (38%) were on insulin therapy (alone or in combination), 54 (54%) were using metformin, and 18 (18%) were using SGLT2 inhibitors. Treatment type did not differ significantly between patients with and without diabetic complications (p > 0.05). Although SGLT2 inhibitors have been shown to reduce skin hyaluronic acid content and may exacerbate xerosis, (10) the potential confounding effect of SGLT2 inhibitor use on ODS scores was not formally adjusted for in the multivariable model due to limited sample size; this should be addressed in future studies.

Sixty-one percent of patients had at least one chronic diabetic complication. Microvascular complications included retinopathy (n = 15, 15%), nephropathy (n = 13, 13%), and peripheral polyneuropathy (n = 35, 35%; plus one patient with autonomic neuropathy). Macrovascular complications included atherosclerotic cardiovascular disease (ASCVD, n = 30), cerebrovascular disease (n = 2), and peripheral artery disease (n = 3). Five patients had a history of diabetic foot. Moisturizer use did not differ significantly based on complication status (p > 0.05).

### 3.2 ODS scores and diabetic complications

Overall ODS score distributions are presented in **Table 2**. All three ODS measures (forearm, lower leg, total) were significantly higher in patients with any chronic complication compared to those without (p = 0.045, p = 0.011, and p = 0.019, respectively; **Table 3**).

**Table 2:** ODS (Overall Dry Skin Score) scores of all patients (n = 100).

	Mean ± SD	Min–max (median)
ODS forearm score	1.03 ± 0.97	0–3 (1)
ODS lower leg score	1.01 ± 0.93	0–3 (1)
ODS total score	2.06 ± 1.70	0–6 (2)

SD, Standard deviation; ODS total score, forearm + lower leg score (range 0–8).

**Table 3:** Comparison of ODS scores according to presence of any chronic complication.

	Complication present — Mean ± SD	Complication present — Min–max (median)	Complication absent — Mean ± SD	Complication absent — Min–max (median)	P
ODS forearm score	1.18 ± 1.01	0–3 (1)	0.79 ± 0.86	0–3 (1)	0.045*
ODS lower leg score	1.20 ± 0.96	0–3 (1)	0.72 ± 0.79	0–3 (1)	0.011*
ODS total score	2.38 ± 1.73	0–6 (2)	1.56 ± 1.55	0–6 (1)	0.019*

*p < 0.05 (Independent Samples t-test). SD: Standard deviation.

The ODS forearm score was significantly higher in patients with retinopathy (p = 0.003) and peripheral polyneuropathy (p = 0.017; **Table 4**). The ODS lower leg score was significantly higher only in patients with retinopathy (p = 0.007; **Table 5**). The total ODS score was significantly higher in patients with retinopathy (p = 0.002), nephropathy (p = 0.047), and peripheral polyneuropathy (p = 0.027; **Table 6**).

**Table 4:** Comparison of ODS forearm scores according to individual complications.

Complication		Mean ± SD	Min–max (median)	P
ASCVD	Present	1.17 ± 0.95	0–3 (1)	0.358
	Absent	0.97 ± 0.98	0–3 (1)	
Retinopathy	Present	1.73 ± 0.96	0–3 (2)	0.003**
	Absent	0.91 ± 0.92	0–3 (1)	
Nephropathy	Present	1.54 ± 1.13	0–3 (2)	0.063
	Absent	0.95 ± 0.93	0–3 (1)	
Peripheral polyneuropathy	Present	1.34 ± 1.06	0–3 (1)	0.017*
	Absent	0.86 ± 0.88	0–3 (1)	

*p < 0.05; **p < 0.01. Independent Samples t-test or Mann–Whitney U test as appropriate. ASCVD, Atherosclerotic cardiovascular disease; SD, Standard deviation.

**Table 5:** Comparison of ODS lower leg scores according to individual complications.

Complication		Mean ± SD	Min–max (median)	P
ASCVD	Present	1.00 ± 0.74	0–3 (1)	0.944
	Absent	1.01 ± 1.00	0–3 (1)	
Retinopathy	Present	1.60 ± 0.91	0–3 (2)	0.007**
	Absent	0.91 ± 0.89	0–3 (1)	
Nephropathy	Present	1.46 ± 1.13	0–3 (1)	0.105
	Absent	0.94 ± 0.88	0–3 (1)	
Peripheral polyneuropathy	Present	1.23 ± 0.97	0–3 (1)	0.083
	Absent	0.89 ± 0.89	0–3 (1)	

**p < 0.01. Independent Samples t-test or Mann–Whitney U test as appropriate. ASCVD, Atherosclerotic cardiovascular disease; SD, Standard deviation.

**Table 6:** Comparison of ODS total scores according to individual complications.

Complication		Mean ± SD	Min–max (median)	P
ASCVD	Present	2.17 ± 1.44	0–6 (2)	0.684
	Absent	2.01 ± 1.81	0–6 (2)	
Retinopathy	Present	3.33 ± 1.63	0–6 (3)	0.002**
	Absent	1.84 ± 1.62	0–6 (2)	
Nephropathy	Present	3.00 ± 1.96	0–5 (4)	0.047*
	Absent	1.92 ± 1.63	0–6 (2)	
Peripheral polyneuropathy	Present	2.57 ± 1.70	0–6 (3)	0.027*
	Absent	1.78 ± 1.65	0–6 (2)	

*p < 0.05; **p < 0.01. Independent Samples t-test or Mann–Whitney U test as appropriate. ASCVD, Atherosclerotic cardiovascular disease; SD, Standard deviation.Bold values indicate statistically significant results (p < 0.05).

### 3.3 Multivariable analysis

In the multivariable linear regression model, only age (β = 0.350, p = 0.002) and retinopathy (β = 0.221, p = 0.042) were independently associated with total ODS score (**Table 1**). The positive beta coefficient for retinopathy indicates that patients with retinopathy had higher total ODS scores compared to those without, consistent with the univariate findings. Nephropathy and peripheral polyneuropathy did not remain significant after adjustment for other variables, suggesting that their univariate associations may be partially mediated by age or shared microvascular burden.

## 4 Discussion

The identification of simple, non-invasive clinical markers that may signal microvascular complications is of particular importance in diabetes management. The ODS score represents a rapid, non-invasive, and low-cost assessment that can be easily applied during routine outpatient visits without the need for laboratory testing or specialized equipment. Unlike traditional metabolic risk markers, skin dryness is a readily observable finding that may prompt clinicians to consider underlying microvascular involvement. Our findings suggest that increased skin dryness, as reflected by higher ODS scores, may serve as an adjunctive clinical signal for diabetic retinopathy, and that incorporating skin assessment into routine diabetes follow-up may support earlier risk stratification and timely referral for ophthalmologic evaluation, particularly in primary care settings where access to specialized screening may be limited.

Skin issues are frequently encountered in individuals with diabetes mellitus, with approximately one-third of patients experiencing some form of skin disorder at some point during their illness. (11–13) The emergence of skin issues in diabetes mellitus is complex and multifactorial, involving changes to microvascular and macrovascular circulation, reduced immunity, and altered collagen synthesis. Factors that increase the risk of skin complications in diabetes include poor glycemic control, duration of diabetes, neuropathy, peripheral vascular disease, obesity, and smoking. (14, 15).

Research has shown that individuals with diabetes mellitus have reduced hydration of the stratum corneum and decreased activity of the sebaceous glands. (16, 17) Xerosis, characterized as abnormal dryness and flaking of the skin, is a common skin condition in these patients, affecting approximately one-quarter of diabetic patients. (18) Decreased sweating due to autonomic neuropathy can result in excessively dry and flaky skin. (19) Moreover, skin dryness, calluses, and cracks contribute to the development of ulcers (20), and diabetic foot wounds may form and progress due to skin cracks. (21, 22).

A relevant parallel exists in the ocular surface literature. Dry eye disease is common in diabetes and has been attributed to degradation of the lacrimal glands. (23) A study of 200 diabetic patients demonstrated a higher prevalence of dry eyes compared to non-diabetic controls, and showed a significant correlation between increasing dry eye severity, decreased corneal nerve sensitivity, and increasing diabetic retinopathy. (24) A positive correlation between dry skin and dry eyes has also been reported in patients with diabetes, (25) suggesting that both reflect common microvascular and neuropathic pathophysiology. Our findings extend this concept to the domain of cutaneous xerosis, demonstrating that objectively measured skin dryness on the forearm and lower leg is consistently elevated in patients with retinopathy.

Our results are consistent with a prior study of 238 young insulin-using diabetic patients, which found that dry, ichthyosiform skin on the anterior legs was significantly associated with diabetic retinopathy (P < 0.0001), and that skin conditions correlated with diabetes duration and the presence of microvascular complications. (26) A more recent investigation utilized a mouse model to explore the relationship between SGLT2 inhibitors and skin dryness, finding a substantial decrease in hyaluronic acid within the skin. (10) This finding suggests that medications commonly prescribed to diabetic patients may exacerbate xerosis, a potential confound that future studies should formally address.

A prospective study of 308 diabetic patients demonstrated a correlation between foot skin dryness assessed by the indicator plaster method (IPM) and an increased likelihood of developing diabetic foot ulcers. (27) Our study revealed a substantially higher total ODS score in individuals with peripheral neuropathy, suggesting that heightened skin dryness may serve as an additional risk factor for leg ulcers. Furthermore, the published literature documents a significant association between severe diabetic foot disease and retinopathy, with shared pathophysiological mechanisms. (28, 29).

While foot xerosis is clinically critical for ulcer prevention, ODS assessment was limited to the forearm and lower leg in the present study to ensure standardized and reproducible scoring. This limits direct comparison with foot-specific tools such as IPM or Neuropad, and should be addressed in future research. Furthermore, although higher ODS scores were associated with diabetic retinopathy, these findings should be interpreted as associative rather than predictive. The clinical appeal of ODS lies in its simplicity and feasibility; however, its role as a screening or risk stratification tool requires prospective validation with longitudinal outcomes.

## 5 Limitations

This study has several important limitations that should be considered when interpreting the findings. The cross-sectional design precludes causal inference and does not allow assessment of the predictive value of ODS for future diabetic complications. The relatively small number of patients with specific complications — particularly retinopathy (n = 15) — limits statistical power; a *post-hoc* analysis indicated approximately 72% power at alpha = 0.05 for this subgroup, below the conventional 80% threshold. The retinopathy findings should therefore be considered hypothesis-generating and require replication in larger, prospective cohorts.

All enrolled patients had type 2 diabetes mellitus; findings may not be generalizable to type 1 diabetes populations, in whom mechanisms of microvascular injury and cutaneous involvement may differ. Furthermore, patients were not stratified by antidiabetic medication class (e.g., oral antidiabetics only, oral antidiabetics plus insulin, or insulin alone), which would have allowed more granular analysis of the relationship between glycemic management strategy, ODS scores, and microvascular complications; future studies should incorporate such subgroup analyses. The ODS is a semi-quantitative ordinal scale, and although appropriate statistical methods were applied, residual measurement subjectivity cannot be excluded. ODS scoring was performed by a single trained assessor; interrater reliability (e.g., Cohen’s kappa or intraclass correlation coefficient) was not formally assessed, which is a limitation for a subjective clinical scale.

Retinopathy severity staging (nonproliferative vs. proliferative) was not available for all patients, precluding dose-response analysis between retinopathy severity and ODS scores. Environmental factors such as seasonality, ambient humidity, medication use (including SGLT2 inhibitors and diuretics), and skincare habits were not systematically recorded and may have influenced skin dryness. Although SGLT2 inhibitor use was documented (18% of patients), its potential confounding effect on ODS scores was not formally adjusted for in the multivariable model. ODS assessment was performed on the forearm and lower leg; therefore, extrapolation to foot-specific outcomes such as ulcer risk should be made with caution. Future prospective, multicenter studies incorporating objective skin hydration measurements and sudomotor assessments are warranted.

## 6 Conclusion

In this study, ODS scores were significantly higher in patients with diabetic retinopathy across all three measurement sites (forearm, lower leg, and total), and retinopathy was the only complication to remain independently associated with total ODS score in multivariable analysis. These findings suggest that patients with high ODS scores in routine clinical evaluation should be prioritized for ophthalmologic retinopathy screening.

All patients diagnosed with diabetes mellitus should be offered appropriate skin moisturization (30) and patients with elevated ODS scores may benefit from timely ophthalmologic evaluation, given that ODS may serve as an adjunctive clinical signal for coexisting microvascular involvement. Although higher ODS scores were associated with diabetic retinopathy, the cross-sectional nature of this study precludes causal or predictive inferences. ODS may serve as a simple clinical indicator of coexisting microvascular involvement; however, prospective studies are required to determine its value in predicting retinopathy onset or progression.

## Data Availability

The original contributions presented in the study are included in the article/supplementary material. Further inquiries can be directed to the corresponding author.
